# Effect of wetland degradation on plant community characteristic and aboveground biomass in the lower reaches of the Yellow River

**DOI:** 10.3389/fpls.2025.1719185

**Published:** 2026-01-19

**Authors:** Yike Huang, Mingli Zhang, Zhiwei Mao, Xinyu Li, Linjun Xu, Jialiang Nie, Xiu Li, Mengxia Li, Wenkai Chen, Junxiang Ding

**Affiliations:** 1 School of Ecology and Environment, Zhengzhou University, Zhengzhou, Henan, China; 2 Chengdu Botanical Garden (Chengdu Park City Botanical Science Research Institute), Chengdu, Sichuan, China; 3 Water Conservancy and Irrigation District Engineering Construction Administration of Xixia Yuan Water Conservancy Project, Department of Water Resources of Henan Province, Zhengzhou, Henan, China

**Keywords:** aboveground biomass, wetland degradation, community characteristics, soil properties, the Yellow River, wetland ecosystems

## Abstract

**Introduction:**

Wetlands are among the most vital ecosystems on Earth, yet they also face severe degradation risks due to intensifying human activities and climate change. Clarifying the response of wetland plant communities to habitat degradation and its key drivers is therefore essential for the effective conservation, restoration, and management of wetland ecosystems. However, how plant community biomass responds to degradation and the underlying drivers remain poorly understood.

**Methods:**

Here, we measured plant community characteristics and soil physicochemical properties along a well-characterized degradation gradient of wetlands in the lower reaches of the Yellow River to determine the drivers of variation in plant community aboveground biomass.

**Results:**

The results showed that aboveground biomass, community coverage, density, and height of the wetland plant community decreased consistently with intensifying degradation, whereas species diversity showed no significant change. The variation in aboveground biomass was significantly associated with community structural metrics (e.g., coverage, density, height) but not with species diversity. Furthermore, changes in soil nutrient availability rather than water conditions were the dominant environmental factor driving the decline in aboveground biomass with wetland degradation.

**Discussion:**

Collectively, these findings underscore the critical role of soil nutrient availability in mediating wetland plant community structure and function under degradation scenarios. This insight is crucial for understanding wetland ecosystem degradation mechanisms and could inform restoration strategies for degraded wetlands.

## 1 Introduction

Wetlands are some of the planet’s most important ecosystems, playing a critical role in maintaining biodiversity, regulating climate, and carbon sequestration (Zedler and Kercher, 2005; Bao et al., 2023). Yet they also represent one of Earth’s most threatened ecosystems. According to recent reports, the world’s wetlands have lost 60, 900 km^2^ over the past forty years due to human activities and climate change (Mao et al., 2025). This degradation has severely impaired wetland ecosystem services essential for human well-being, including altered habitat conditions (e.g., hydrology, nutrient cycling) and plant community restructuring (e.g., species turnover, diversity decline) (Wan et al., 2024; Xu et al., 2024). As a proxy for ecosystem structure and functions, plant productivity is intrinsically linked to species composition, diversity, and functional trait expression (Xu et al., 2024). Therefore, elucidating the response patterns of biomass production to degradation and their underlying drivers is crucial for understanding the adaptation strategies of wetland plant communities. This knowledge will scientifically underpin wetland conservation and restoration practices.

Extensive research has focused on wetland ecosystem structure and function under environmental change. However, the responses of plant community biomass to degradation remain inconsistent, with decreasing (Zhang et al., 2019), increasing (Shang and Yang, 2012), and non-significant changes (Xu et al., 2024) in aboveground biomass reported under progressive degradation. These contrasting patterns can likely be attributed to disparities in community characteristics and biotic factors among different wetland types. For example, alpine wetlands on the Qinghai-Tibet Plateau exhibit declines in plant height, coverage, and density with progressive degradation (Xu et al., 2024); in contrast, salt marsh wetlands on Songnen Plain show initial decreases in these characteristics followed by subsequent increases (Wan et al., 2024). Species diversity, a key determinant of ecosystem productivity, also exhibits highly variable responses to wetland degradation. For example, some studies have reported decreases in diversity with degradation (Eneyew and Assefa, 2021; Nyandwi and Ndikubwimana, 2024), while others have observed increase (Thakur et al., 2017; Shi et al., 2020) or non-significant changes (Ren et al., 2015; Wan et al., 2024). This diverse variation in community characteristics could amplify the uncertainty in predicting biomass responses and their underlying drivers during wetland degradation.

In addition to biotic factors, degradation-induced changes in soil properties can significantly alter plant community structure and ecosystem functions (Zhang et al., 2019; Han et al., 2020; Wu et al., 2024). Generally, soil environments (e.g., bulk density, salinity and moisture), soil nutrient availability and soil microbial communities undergo significant changes along with wetland degradation (Zhang et al., 2019; Chen et al., 2025b). These changes in soil factors may directly (e.g., plant establishment) or indirectly (e.g., growth performance) affect the biomass production of the wetland ecosystem. However, the responses of soil physicochemical properties to wetland degradation are not always consistent across different regions and wetland types. For instance, a recent study on inland salt‐marsh wetlands demonstrated a marked reduction in soil nutrient availability (e.g., carbon, nitrogen, and phosphorus) with progressive degradation (Wan et al., 2024), whereas research in alpine wetlands indicated that soil moisture and salinity changes predominate during degradation (Xu et al., 2024). This context-dependent variation in soil properties may further drive the inconsistent plant community and biomass responses observed across the degradation gradient in wetlands. Indeed, recent studies have uncovered divergent drivers underlying community characteristics across ecosystems. For instance, Wan et al. (2024) identified declining soil N and P and increasing C:N ratio as primary controls on vegetation structure (e.g., plant cover, density, height) and diversity under degradation; in contrast, Gerdol et al. (2018) showed that soil moisture governed community structure, while Wang et al. (2023) identified salinity and electrical conductivity as primary drivers of plant diversity. Therefore, the dominant factors controlling the responses of plant community biomass to degradation may vary among wetland types and environmental conditions.

In the lower reaches of the Yellow River, abundant riparian wetlands have developed along both riverbanks due to sustained recharge of groundwater and surface water (Hong et al., 2021). These wetlands perform critical ecological functions including water conservation, climate regulation, water purification, and biodiversity protection. However, intensive human activities and environmental changes have led to significant contraction and degradation of the riparian wetlands in the lower reaches of the Yellow River (He et al., 2015; Dou et al., 2025). This makes the riparian wetlands in the lower reaches of the Yellow River a suitable experimental platform for exploring the adaptation and driving factors of wetland plant community structure and function under degradation. However, current studies on the riparian wetlands in the lower reaches of the Yellow River mainly focus on vegetation dynamics (He et al., 2015), elemental transport (Chen et al., 2005; Li et al., 2017) and eco-hydrological patterns (Wang et al., 2006; Xu et al., 2016), and fewer studies have investigated plant community changes and their influencing factors from a degradation perspective. As a result, our understanding of the variation patterns and key driving factors of plant communities and biomass along the degradation gradient in the Yellow River riparian wetlands remains limited. This knowledge gap is of critical importance for unraveling the processes and mechanisms of wetland degradation. Here, based on long-term field investigations on soil environment and plant community characteristics, we selected nine study sites in the wetland under varying degree of degradation (i.e., light, moderate, and severe degradation) in the Henan section of the lower reaches of the Yellow River. By examining changes in plant community characteristics (e.g., species composition, diversity, coverage, and aboveground biomass) and soil physicochemical properties (e.g., soil water content, pH and nutrients) along the degradation gradient, we aimed to clarify the response of community aboveground biomass to wetland degradation and its environmental drivers. Specifically, we address two questions: (1) how does wetland degradation alter community characteristics and aboveground biomass? (2) What are the determinants of variation in community aboveground biomass along degradation gradients? Resolving these questions will help clarify the adaptation mechanisms of wetlands to climate change and human pressures, thereby guiding effective conservation and restoration of degraded ecosystems.

## 2 Materials and methods

### 2.1 Study area

**Figure 1:**
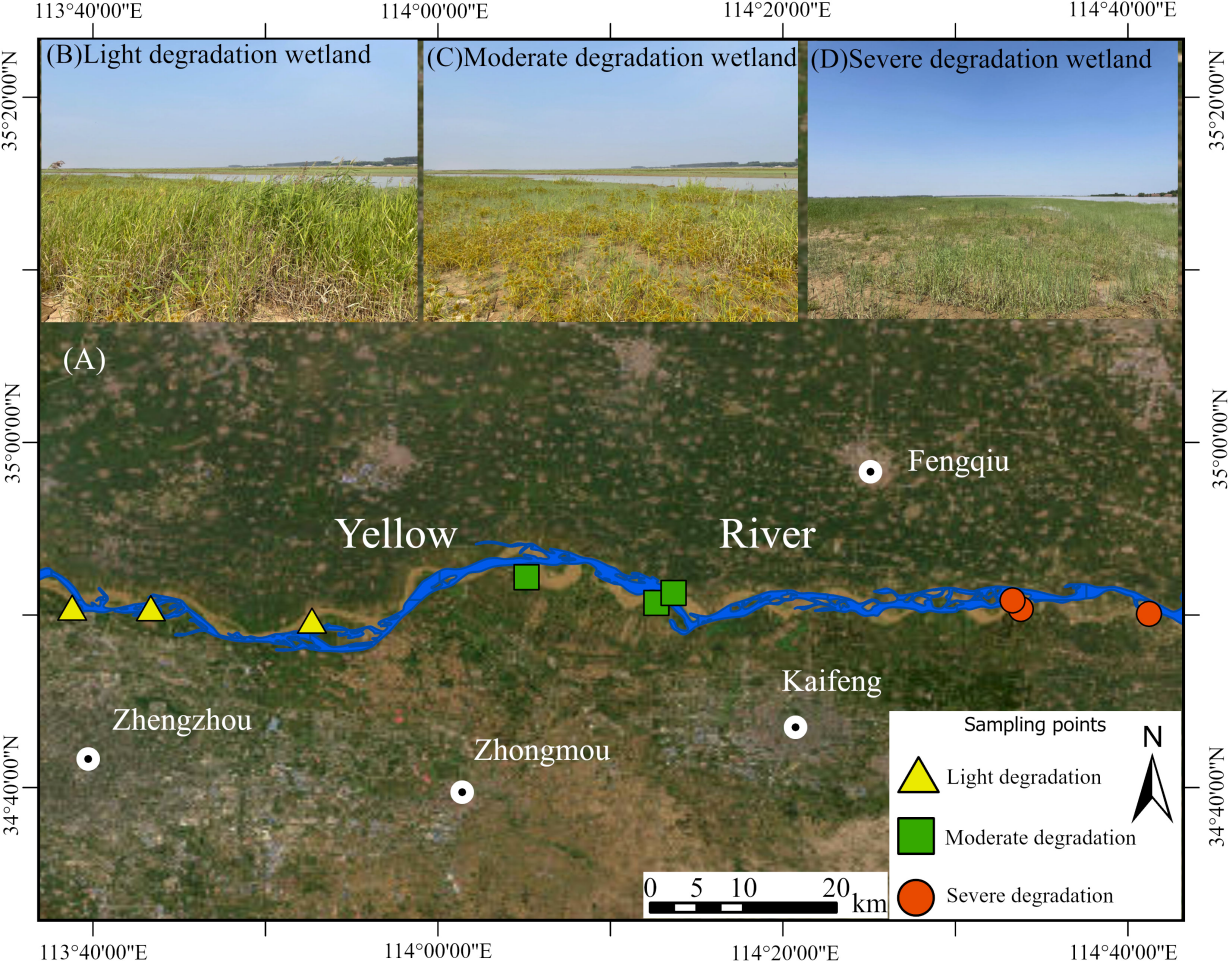
The sampling sites **(A)** in the lower reaches of the Yellow River located in Henan province, China. The nine sampling sites contain **(B)** light, **(C)** moderate and **(D)** severe degradation wetlands.

The study area is located in the reach from Huayuankou to Jiahetan in the lower reaches of the Yellow River, Henan Province, China (34.89°–34.92° N, 113.64°–114.70° E) (**Figure 1**). This region features a temperate continental monsoon climate, with an average annual precipitation of 500 to 800 mm, concentrated primarily between June and September. The mean annual temperature ranges from 12 to 16°C. The study area consists of floodplain wetlands on both sides of the Yellow River, formed by seasonal inundation and located within the low-lying areas of the floodplain. The plant community is dominated by perennial herbs (e.g., *Phragmites australis*, *Calamagrostis pseudophragmites*, *Miscanthus sacchariflorus*, *Typha latifolia*) and annual herbs (e.g., *Aster subulatus*, *Conyza canadensis*, *Potentilla supina*), along with other common species such as *Cyperus glomeratus* and *Alternanthera philoxeroides*. The soils in this area developed primarily from recent river sediments and are classified as alluvial soil (Li et al., 2021).

### 2.2 Plot establishment and sample collection

Prior to sampling, we conducted a three-year investigation (2021-2023) into the geographical distribution of wetlands in the lower reaches of the Yellow River. Based on the data obtained regarding community coverage and soil moisture conditions, we selected nine study sites representing varying degrees of degradation from the surveyed wetlands (Han et al., 2020; Xu et al., 2024). These experimental sites were located 5–10 meters from the river channel and were characterized by similar hydrological conditions. Specifically, we classified the riparian wetlands into light degradation, moderate degradation, and severe degradation according to the vegetation coverage and moisture condition. Lightly degraded wetlands, located within a nature reserve, benefit from a stable water supply, which supports the highest soil water content (25.25-44.76%) and vegetation coverage (54.44%–78.89%). In contrast, moderately degraded wetlands suffer from an unstable water supply, leading to significantly lower values for both soil water content (12.99-31.65%) and vegetation coverage (32.56%–45.00%). Conversely, severely degraded wetlands recorded the lowest levels of soil water content (7.14-25.90%) and vegetation coverage (17.00%–23.33%). Additionally, with the intensification of degradation, the abundance of *Aster subulatus* decreased, the relative abundance of *Cyperus glomeratus* showed no significant change, while that of *Phragmites australis* tended to increase (**Supplementary Figure S1**).

During the peak growing season (August 2023), we established three to five plots (10 m × 10 m) with intervals of over 20 m at each site and recorded their geographic coordinates (latitude, longitude, and elevation). Within each large plot, three 1 m × 1 m quadrats were arranged diagonally for detailed plant community surveys, including measurements of species composition, coverage, and height, as well as for sample collection. Specifically, for each species within the quadrats, we randomly selected five healthy individuals, measured their height, and calculated the mean height. Plant density was determined by counting all individuals within each 1×1 m quadrats. The Shannon-Wiener index was calculated based on species richness and relative abundance. Community coverage was determined by visual estimation within each quadrat. Aboveground biomass was determined by clipping all vegetation at ground level within each 1×1 m quadrat, followed by oven-drying the samples at 65°C to constant mass and recording the dry weight. Subsequently, five soil cores were randomly collected from each experimental plot using a soil auger (5 cm in diameter, 20 cm in length) and thoroughly mixed to form a composite sample.

### 2.3 Determination of soil physicochemical properties

The soil samples were sieved through a 2 mm mesh and divided into two aliquots: one was stored at -20°C for subsequent analysis of soil water content (SWC) and inorganic nitrogen (IN), while the other was air-dried, homogenized, and used for measuring pH, electrical conductivity (EC), total carbon (TC), total nitrogen (TN), available phosphorus (AP) and total phosphorus (TP). SWC was determined using the gravimetric method by oven-drying samples at 65°C for 72 hours until constant mass was achieved. Soil bulk density (BD) was quantified using the ring-knife method (Al-Shammary et al., 2018). IN (i.e., nitrate and ammonium) concentrations were measured in the solution extracted from soil samples with 2 M KCl using a continuous flow analyzer (Autoanalyzer 3; Seal Analytical, GmbH Germany). Soil pH and EC were determined in a 1:2.5 (w/v) soil-water suspension using a glass electrode (model PHS-2, INESA Instrument). TC and TN were analyzed using an elemental analyzer (Vario EL III; Elementar, Langenselbold, Germany). TP concentration was determined by inductively coupled plasma-atomic emission spectrometry (Optima 5300 DV, Perkin Elmer) following acid digestion of soil samples with H_2_SO_4_ and HClO_4_. AP concentration was determined by molybdenum blue colorimetry after extraction with 0.5 M NaHCO_3_.

### 2.4 Statistical analysis

Prior to statistical analyses, data normality and homogeneity of variances were assessed using the Shapiro-Wilk and Levene’s tests, respectively, with log-10 transformation applied whenever necessary. One-way analysis of variance (ANOVA) was used to examine differences in aboveground biomass, species richness, Shannon-Wiener index, coverage, average height, and plant density among wetlands with varying degradation levels. Pearson correlation analysis was conducted to explore relationships between aboveground biomass and plant community characteristics as well as soil physicochemical properties. Redundancy analysis (RDA) was performed to investigate the influence of soil factors (including soil water content, pH, electrical conductivity, and nutrients) on plant community characteristics. Relative importance analysis was conducted to quantify the contributions of soil physicochemical properties and plant community characteristics to the variation in aboveground biomass. Furthermore, Structural equation modeling (SEM) was conducted to examine the direct and indirect pathways through which various influencing factors affect aboveground biomass. All data were initially processed using Excel 2019, with statistical analyses and figure generation performed by Origin 2024, R 4.4.3, and ArcGIS Pro 2024 software.

## 3 Results

### 3.1 Plant community and soil properties in degraded wetlands

**Figure 2:**
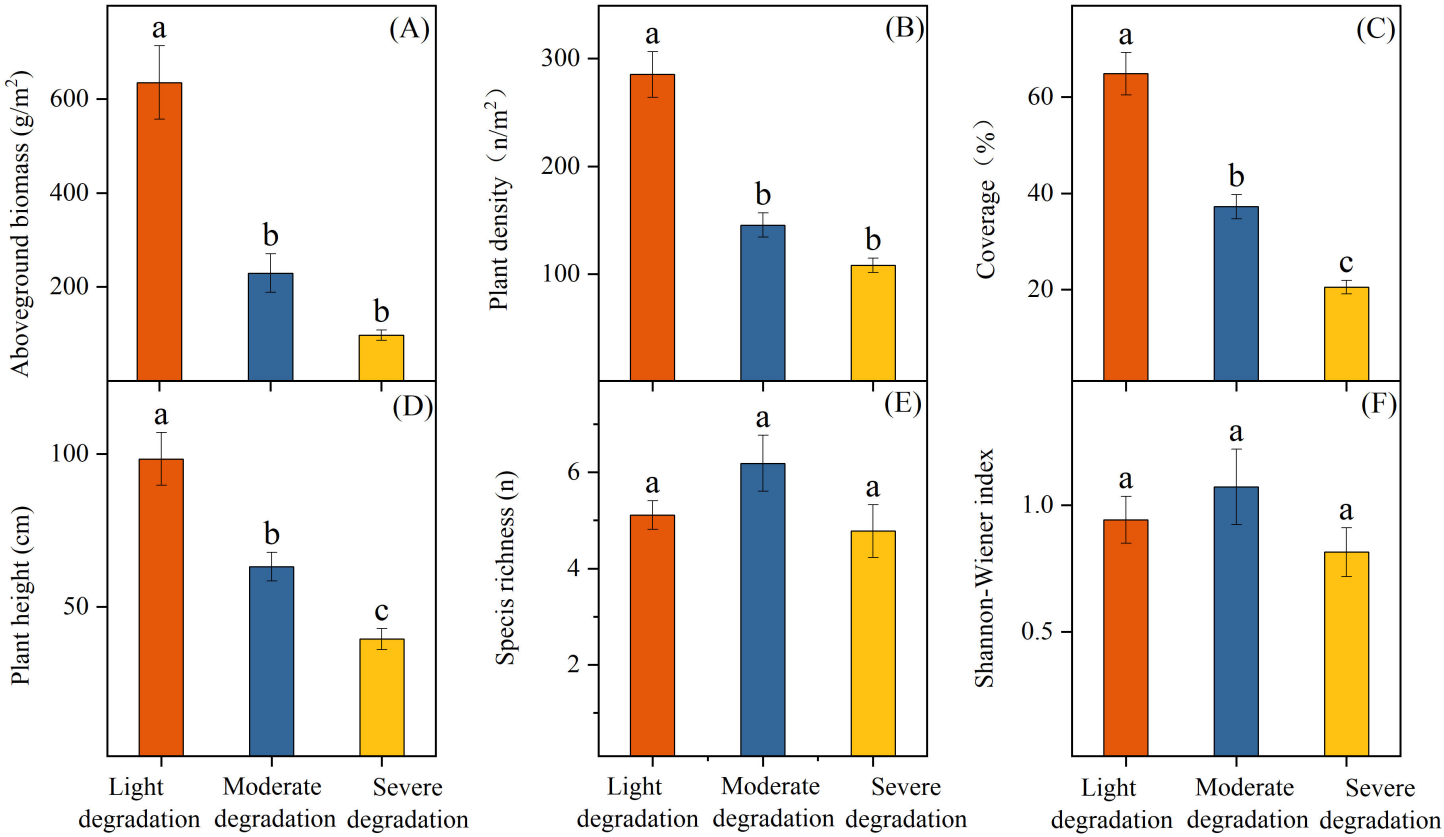
Differences in plant community aboveground biomass **(A)**, plant density **(B)**, coverage **(C)**, plant height **(D)**, species richness **(E)**, and Shannon-Weiner index **(F)** under different degradation levels. Different letters given on the top of vertical bars indicate significant differences among wetlands with different degrees of degradation at *P* < 0.05 levels.

Plant community characteristics differed significantly across wetlands with varying degrees of degradation (*P* < 0.05, **Figures 2A–D**). Specifically, lightly degraded wetlands exhibited the highest values in aboveground biomass (635.05 g/m²), plant density (285.15 n/m²), coverage (64.85%), and mean height (98.42 cm). Moderately degraded wetlands showed intermediate values for these parameters (229.55 g/m², 145.37 n/m², 37.22%, and 63.04 cm, respectively), while severely degraded wetlands had the lowest values (97.27 g/m², 108.07 n/m², 20.48%, and 39.37 cm). In contrast, species richness and Shannon-Wiener index had no significant changes along the degradation gradient (**Figures 2E, F**), with species richness ranging from 3 to 9 n/m² and Shannon-Wiener index varying between 0.44 to 1.75. Soil physicochemical properties also showed significant differences among wetlands under varying degradation degrees. All wetland soils were alkaline, with pH values ranging from 8.21 to 8.58. The highest pH (8.48) occurred in severely degraded wetlands, significantly exceeding values in lightly (8.27) and moderately (8.42) degraded wetlands. Both soil EC and BD increased with degradation severity, while SWC decreased from 35.60% in lightly degraded wetlands to 22.03% and 18.02% in moderately and severely degraded wetlands, respectively. Lightly degraded wetlands had significantly higher soil nutrient contents than moderately and severely degraded wetlands, with the highest values for soil TC (26.23 g/kg), TN (0.88 g/kg), IN (60.46 mg/kg), and AP (17.60 mg/kg) (**Table 1**).

**Table 1:** Changes in soil physical and chemical properties under different degradation degrees (mean ± standard error).

Degree of degradation	Soil pH	Soil electrical conductivity/μS cm^-1^	Soil water content/%	Soil bulk density/g cm^-^³	Soil total C/g kg^-1^	Soil total N/g kg^-1^	Soil inorganic nitrogen/mg kg^-1^	Soil total phosphorus /g kg^-1^	Soil available phosphorus/mg kg^-1^
Light degradation	8.27 ± 0.03a	222.90 ± 42.63a	35.60 ± 6.38a	1.39 ± 0.05a	26.23 ± 3.43a	0.88 ± 0.14a	60.46 ± 3.78a	0.59 ± 0.03a	17.60 ± 4.89a
Moderate degradation	8.42 ± 0.09a	313.93 ± 53.69a	22.03 ± 6.28b	1.56 ± 0.04b	20.44 ± 2.97b	0.55 ± 0.08b	50.13 ± 6.02b	0.56 ± 0.05ab	10.00 ± 3.72b
Severe degradation	8.48 ± 0.07b	363.33 ± 45.54b	18.02 ± 6.83b	1.61 ± 0.03c	16.19 ± 0.98c	0.40 ± 0.08c	44.63 ± 4.97b	0.61 ± 0.04b	9.24 ± 3.13b

Different lowercase letters in the same column indicate significant differences under different degrees of degeneration (P < 0.05).

### 3.2 Effects of abiotic and biotic factors on aboveground biomass

**Figure 3:**
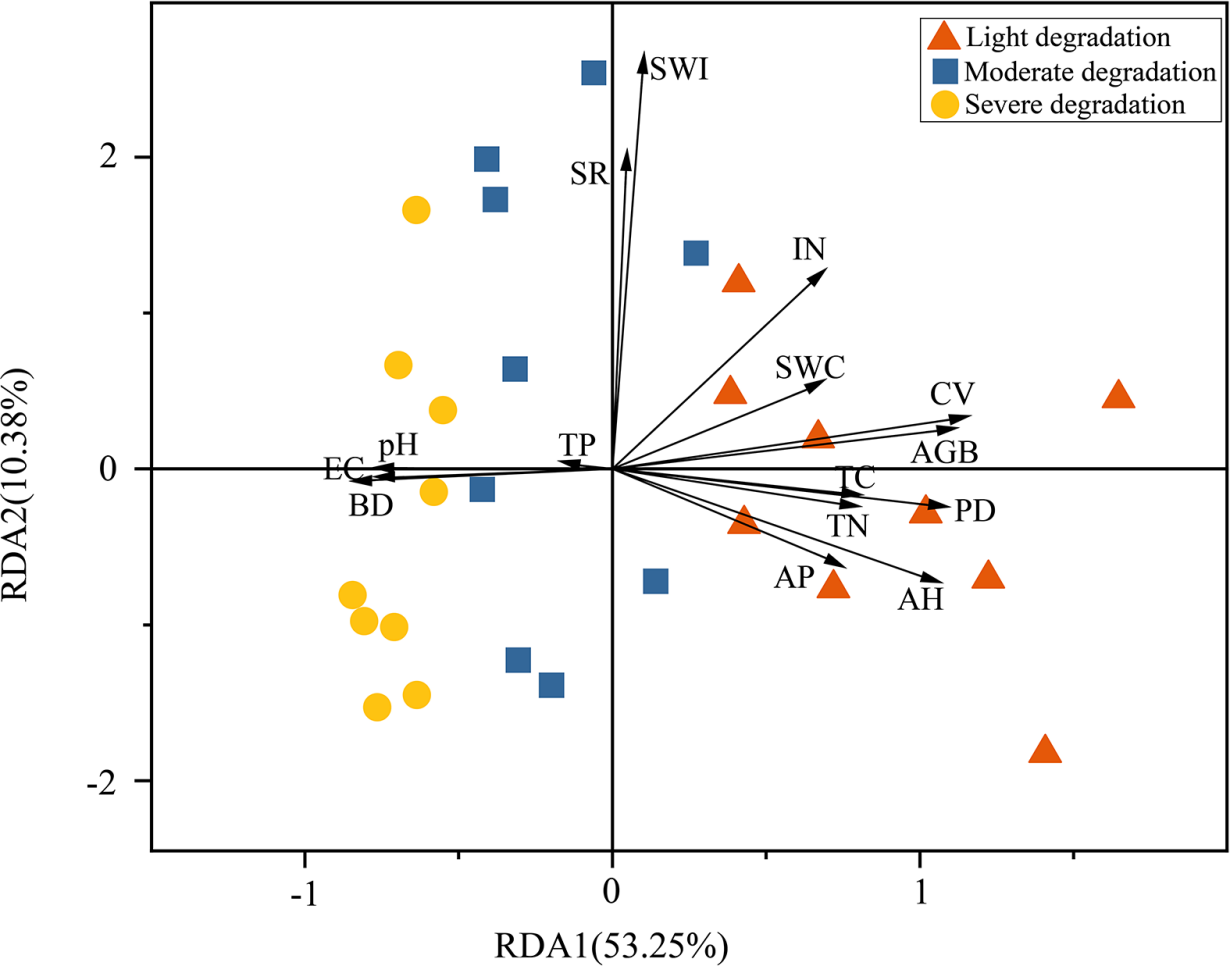
Redundancy analysis of soil physicochemical properties and plant community characteristics under different degradation levels. AGB, above-ground biomass; SWC, soil water content; pH, soil pH; TC, total soil carbon; TN, total soil nitrogen; IN, soil inorganic nitrogen; TP, total soil phosphorus; AP, soil available phosphorus; BD, soil bulk density; EC, Soil electrical conductivity; SR, species richness; SWI, Shannon-Weiner Index; CV, plant coverage; PD, plant density; AH, average plant height.

**Figure 4:**
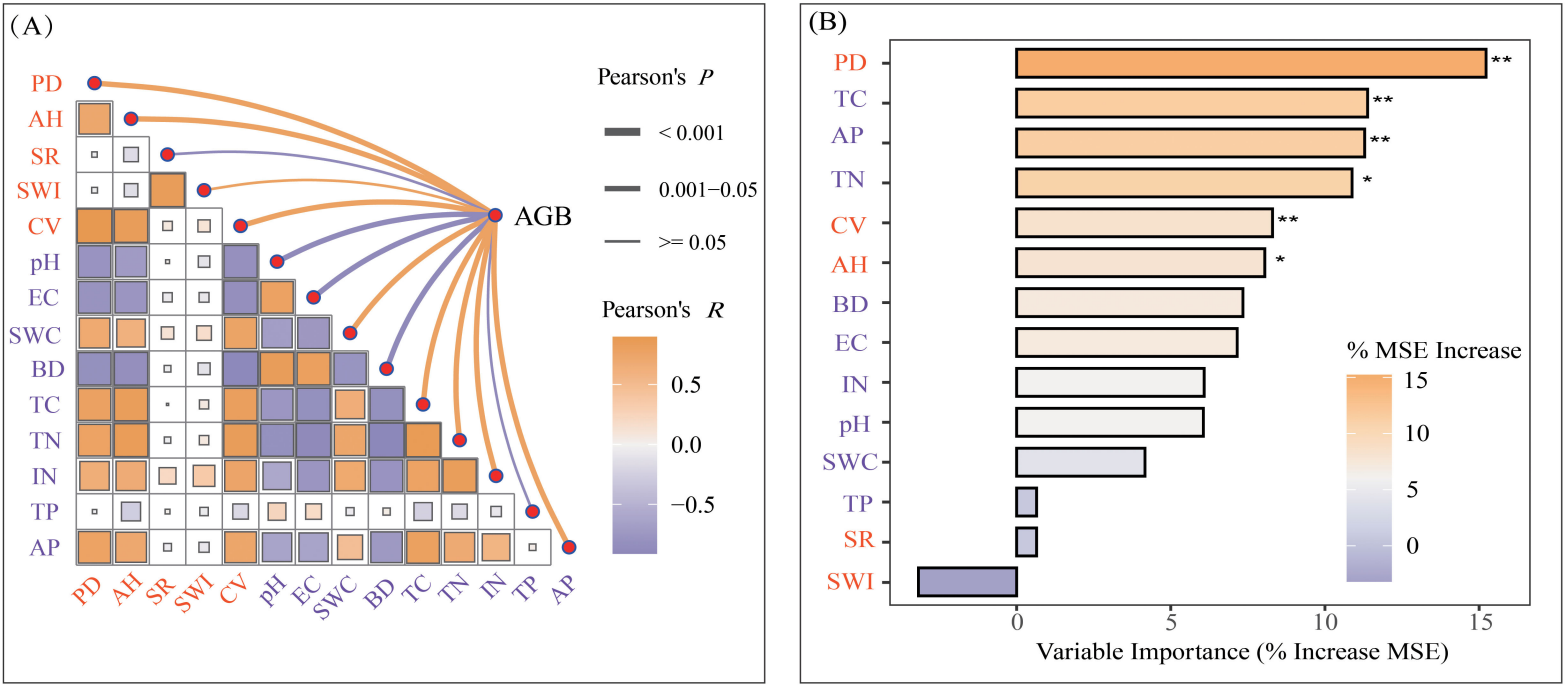
Correlation between aboveground biomass, plant community characteristics, and soil physicochemical properties under different degrees of degradation **(A)** and relative importance analysis of soil physicochemical properties and plant community characteristics on aboveground biomass in wetlands of the lower reaches of the Yellow River **(B)**. Pearson’s *P* denotes the *P* value of Pearson’s correlation coefficient; Pearson’s *R* denotes the Pearson correlation coefficient. AGB, aboveground biomass; PD, plant density; AH, average plant height; SR, species richness; SWI, Shannon-Wiener Index; CV, plant coverage; pH, soil pH; EC, soil electrical conductivity; SWC, soil water content; BD, soil bulk density; TC, total soil carbon; TN, total soil nitrogen; IN, soil inorganic nitrogen; TP, total soil phosphorus; AP, soil availablephosphorus. *P ≤ 0.05; **P ≤ 0.01.

**Figure 5:**
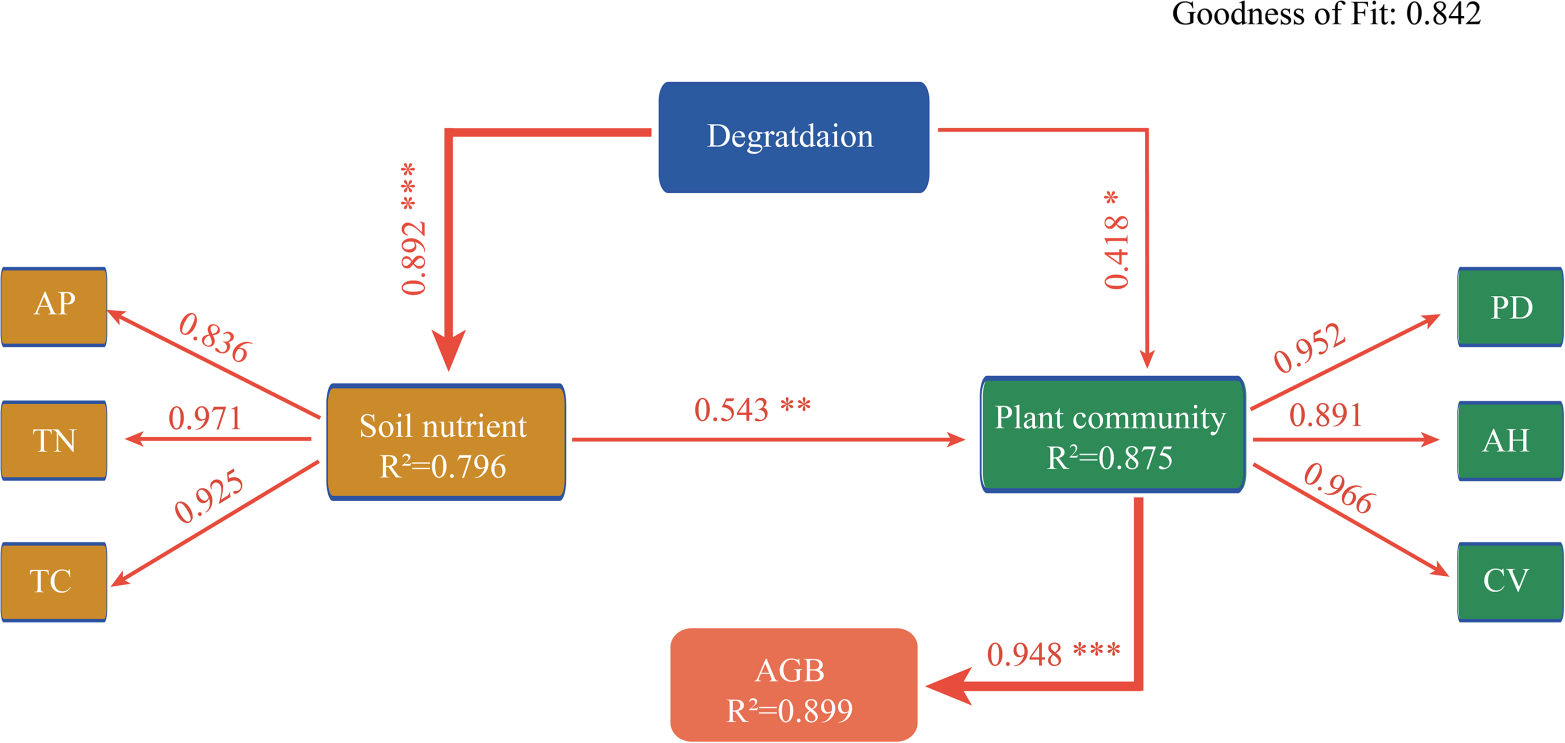
Structural equation model diagram of the pathways through which plant community characteristics and soil physicochemical properties affect aboveground biomass under different degradation degrees. Red line represents positive effect. R^2^ indicates the variance of model. Values on the line are the standard path coefficients. Goodness of fit = 0.842. *P ≤ 0.05; **P ≤ 0.01; ***P ≤ 0.001.

Redundancy analysis (RDA) showed significant relationships between wetland plant community characteristics and soil physicochemical properties (**Figure 3**). Specifically, the first two RDA axes collectively explained 63.63% of the total variation in plant community characteristics, with the first axis contributing 53.25% and the second axis accounting for 10.38%. Plant community parameters including aboveground biomass, density, coverage, and mean height showed positive correlations with soil TC, TN, AP, and IN, yet negative correlations with soil pH, EC, and BD. However, no significant correlations were observed with species richness or the Shannon-Wiener index (**Figure 3**). Correlation analysis further showed that aboveground biomass, plant density, coverage, and mean height were positively correlated with SWC, TC, TN, IN, and AP, but negatively correlated with soil pH, EC, and BD. Aboveground biomass showed significant positive correlations with plant density, coverage, and mean height, but no significant relationships with species richness or the Shannon-Wiener index (**Figure 4A**). Furthermore, relative importance analysis identified plant density as the predominant biotic determinant of aboveground biomass, while TC, AP, and TN emerged as the key edaphic drivers. SWC, pH, and EC exhibited relatively weaker influences on aboveground biomass (**Figure 4B**). SEM revealed that the effect of degradation-induced soil nutrient changes on aboveground biomass was primarily mediated by plant community characteristics, namely coverage, density, and height (**Figure 5**).

## 4 Discussion

### 4.1 Influences of wetland degradation on aboveground biomass and plant community characteristics

Habitat degradation typically reshapes plant communities by altering their species composition, diversity, cover, and density, which may subsequently mediate biomass production through impacts on plant establishment and growth (Weigelt et al., 2008; Moor et al., 2017). Our results demonstrated a consistent decline in aboveground biomass along the degradation gradient (**Figure 2A**), consistent with findings from inland salt marsh wetland, alpine wetlands, tussock meadow wetlands, and forest ecosystems (Zhang et al., 2019; Zhou et al., 2021; Zhang et al., 2022; Wan et al., 2024: Xu et al., 2024). This pattern in community biomass reinforces the previous perspective that diminished productivity is a pivotal indicator for identifying the degradation status of wetland ecosystems (Wang et al., 2019; Daleo et al., 2023; Ding et al., 2025a). Species diversity is widely recognized as a critical driver of biomass production, as it can enhance community productivity through mechanisms such as niche complementarity or the insurance effect (Craven et al., 2018). Global studies across forest and grassland ecosystems have demonstrated a positive effect of biodiversity on ecosystem productivity (Cardinale et al., 2007; Liang et al., 2016). However, in our study, neither species richness nor Shannon-Wiener index showed significant changes with increasing degradation levels, and no significant correlation was observed between species diversity indices and aboveground biomass of plant communities (**Figures 2E, F**; **Figure 4A**). These findings suggest that species diversity plays a relatively minor role in driving variations in plant community biomass within our studied wetland ecosystem. This is likely due to the consistently low species richness (mean: 5.8 n/m²; range: 3–9 n/m²) in our study area, which constrains the potential for niche complementarity effects (Wang et al., 2019; Ding et al., 2025b). Previous studies have found that positive effects of species diversity on ecosystem productivity mostly occur in regions with high species richness (Cardinale et al., 2007; Liang et al., 2016). Moreover, we observed that plant communities across degradation gradients exhibited similar species compositions, with *Phragmites australis*, *Calamagrostis pseudophragmites*, and *Aster subulatus* dominating all sites (relative abundance >90%). This suggests that changes in aboveground biomass under degradation are likely regulated by biotic mechanisms other than species diversity, such as plant density, cover, and height.

It is noteworthy that plant community density and coverage consistently declined with intensifying degradation (**Figures 2B, C**). The reduction in density and coverage indicates a self-thinning adaptation to habitat degradation, thereby constraining community biomass production (Pavel et al., 2023; Long et al., 2024). Furthermore, community height decreased with increasing degradation (**Figure 2D**). Plant height is closely correlated with leaf and root traits, and together they represent a plant’s integrated strategy for resource acquisition and environmental adaptation (Maynard et al., 2022; Liu et al., 2023; Suo et al., 2024). Generally, taller plants exhibit acquisitive traits, such as high specific leaf area and rapid photosynthetic rates, which are conducive to high biomass production (Maynard et al., 2022; Liu et al., 2023; Suo et al., 2024). Our prior research in this region also revealed a shift in wetland plant community traits from an acquisitive (high SLA, high nitrogen concentration) to a conservative (high leaf dry matter content) strategy under degradation (Ding et al., 2025a). Consequently, the adjustment of plant traits and resource strategies associated with the reduction in community height is also a critical pathway leading to biomass decline. Overall, our findings demonstrate that the biomass reduction in degraded wetlands of the lower reaches of the Yellow River is unrelated to shifts in species diversity but is primarily driven by alterations in community structure (e.g., coverage, density, and height) and associated growth strategies.

### 4.2 Environmental drivers of aboveground biomass along degradation gradients

In addition to biotic factors, wetland degradation is typically accompanied by changes in habitat conditions (e.g., soil moisture and nutrient availability). In this study, soil moisture and nutrient availability decreased significantly with increasing degradation degree, whereas soil pH, EC, and BD increased significantly (**Table 1**). These findings align with prior studies, suggesting that reduced soil resource availability and intensified salinization may serve as key indicators of wetland degradation (Gerdol et al., 2018; Liu et al., 2020; Wan et al., 2024). Riparian wetlands in the lower reaches of the Yellow River typically occur in narrow zones adjacent to riverbanks, which makes them highly sensitive to hydrological fluctuations (He et al., 2015). Furthermore, soil nutrients in these ecosystems are predominantly controlled by river overflow regimes (Liang and Ding, 2004; Meng et al., 2017). Consequently, soil moisture availability likely governs aboveground biomass variation through its dual regulation of plant water stress and nutrient availability. However, relative importance analysis demonstrated that soil nutrients exerted a stronger influence than soil moisture on aboveground biomass variability (**Figure 4B**). This unexpected result may stem from two reasons. First, wetland plants differ in their adaptation to hydrological fluctuations and nutrient availability. In our study area, dominant species such as *P. australis* and *C. pseudophragmites* exhibit strong tolerance to variations in soil moisture, particularly *P. australis*, whose habitat can range from desert with extreme water shortage to the flooded wetland environment (Packer et al., 2017). Second, the observed pattern may be due to differences in the magnitude of limitation and the heterogeneity of soil moisture and nutrients in the study area. Since the implementation of the Water-Sediment Regulation Scheme in 2002, the recharge of water and nutrient to riparian wetlands via surface runoff has been substantially reduced (Chu, 2014; Xu et al., 2016). Crucially, while water deficits can be alleviated by groundwater recharge, nutrient supply remains dependent on diminished hydrological processes. This could result in soil nutrient availability being more limited and heterogeneous than that of water, thus constitute a more critical factor controlling aboveground biomass accumulation.

Soil nutrients critically regulate plant growth and community structure dynamics. And a decrease in soil nutrient availability generally has a negative effect on wetland plant community height, density, and coverage (Wan et al., 2024). Indeed, we observed synchronous declines in community plant density, coverage, and height with decreasing soil nutrient availability along the degradation gradient (**Figures 3**, **4**). As these variables are major determinants of aboveground biomass, our findings suggest that soil nutrient limitation likely constrains biomass accumulation by suppressing both individual plant growth and population establishment (**Figure 5**). Meanwhile, our prior study revealed that as degradation intensifies, leaf and root traits of wetland plant communities shift from acquisitive (e.g., high specific leaf area, leaf and root nitrogen concentration) to conservative (e.g., high leaf dry matter content and root tissue density) (Ding et al., 2025a). These plant trait adjustments shape community structure dynamics, which in turn mediates aboveground biomass production in response to nutrient availability. Additionally, the dominant role of soil nutrient availability on aboveground biomass identified in this study may reflect a carbon-nutrient trade-off mechanism, whereby under degraded conditions, plants need to reallocate limited carbon resources to optimize nutrient acquisition efficiency (Chen et al., 2025a). That is to say, in addition to specific root morphological traits, wetland plants may adapt to limited soil nutrients by adjusting their belowground biomass allocation, such as constructing more fine roots to enhance nutrient acquisition. The importance of such root biomass adjustments is increasingly recognized (Ma et al., 2021; Pan et al., 2024), and their omission may underlie the largely unexplained variation in community biomass along the degradation gradient observed here. Therefore, future studies should integrate belowground biomass and unconsidered surrounding environmental conditions, such as the intensity of human activities, to advance our mechanistic understanding of wetland ecosystem degradation processes.

## 5 Conclusions

By investigating plant community characteristics, aboveground biomass, and soil properties along a degradation gradient in the lower reaches of the Yellow River, this study explored the biotic and abiotic drivers and underlying mechanisms of variation in wetland plant community aboveground biomass. Our results showed that wetland degradation significantly reduced plant coverage, density, height, and aboveground biomass, with no significant effect on species diversity. Soil nutrient availability was the dominant environmental driver of variation in plant community characteristics and aboveground biomass, primarily regulating aboveground biomass by changing community density, height, and cover rather than species diversity. Overall, these findings reveal variation patterns and drivers of wetland plant community characteristics and aboveground biomass under degradation gradients, highlighting the critical role of biotic-abiotic interactions in mediating wetland ecosystem functions. These insights are critical for understanding mechanisms maintaining wetland ecosystem stability and will directly inform restoration strategies for degraded wetlands.

## Data Availability

The data that support the findings of this study are available from the corresponding author upon reasonable request.
