# Supplementary material for: Effect of wetland degradation on plant community characteristic and aboveground biomass in the lower reaches of the Yellow River
Source: Front Plant Sci. 2026 Jan 19;16:1719185. doi: 10.3389/fpls.2025.1719185 (PMC12862070; doi:10.3389/fpls.2025.1719185)
Supplement: Supplementary file 1 [file SupplementaryFile1.docx]

**Supplementary Figure S1** Species relative abundance in different degradation wetlands. Abbreviations: L, lightly degraded wetland; M, moderately degraded wetland; S, severely degraded wetland.
